# Induction of T‐Cell Differentiation by KLF4 in T‐Cell Acute Lymphoblastic Leukemia Cells Harboring Activating Mutation in NOTCH3

**DOI:** 10.1096/fj.202402997R

**Published:** 2025-05-12

**Authors:** Mina Noura, Takahiko Yasuda, Hitoshi Kiyoi, Fumihiko Hayakawa

**Affiliations:** ^1^ Division of Cellular and Genetic Sciences, Department of Integrated Health Sciences Nagoya University Graduate School of Medicine Nagoya Japan; ^2^ Clinical Research Center National Hospital Organization Nagoya Medical Center Nagoya Japan; ^3^ Department of Hematology and Oncology Nagoya University Graduate School of Medicine Nagoya Japan

**Keywords:** cell differentiation, KLF4, leukemia, NOTCH3, T‐ALL

## Abstract

Krüppel‐like factor 4 (KLF4) exhibits both oncogenic and tumor‐suppressive effects depending on the type of cancer and cellular context. In T‐cell acute lymphoblastic leukemia (T‐ALL), *KLF4* expression is silenced by promoter methylation, and the induction of KLF4 suppresses the proliferation of T‐ALL cells. Therefore, KLF4 is thought to function as a tumor suppressor in T‐ALL cells; however, its role in the differentiation of T‐ALL cells remains unclear. Here, we show that KLF4 induced T‐cell differentiation and apoptosis in TALL‐1 cells harboring an activating mutation in NOTCH3. Mechanistically, KLF4 directly downregulated *NOTCH3* expression by binding to its promoter, thereby promoting the differentiation of CD4/CD8 double‐positive cells into CD4 single‐positive cells, with the differentiated cells subsequently undergoing apoptosis. Furthermore, we found that APTO‐253, a small‐molecule inducer of KLF4, effectively suppressed cell growth in TALL‐1 cells by promoting T‐cell differentiation followed by apoptotic cell death. These findings suggest a promising strategy for developing novel differentiation therapies for T‐ALL with NOTCH3 mutations.

## Graphical abstract

KLF4‐induced T‐cell differentiation and apoptosis in TALL‐1 cells harboring an activating mutation in NOTCH3. KLF4 directly downregulated *NOTCH3* expression by binding to its promoter, thereby promoting the differentiation of CD4/CD8 double‐positive cells into CD4 single‐positive cells, with the differentiated cells subsequently undergoing apoptosis. Furthermore, pharmacological induction of KLF4 using APTO‐253, a small‐molecule inducer of KLF4, promoted T‐cell differentiation and exhibited anti‐leukemic activity.

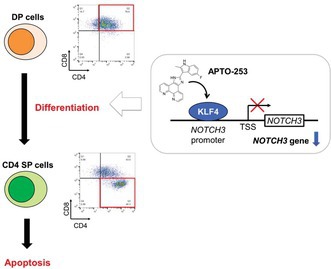

## 1 Introduction

T‐cell acute lymphoblastic leukemia (T‐ALL) is an aggressive malignancy characterized by differentiation arrest and accumulation of immature thymocytes, generally resulting in a less favorable prognosis than most cases of B‐ALL [1]. The recent advent of immunotherapies, including monoclonal antibodies and chimeric antigen receptor T cells that target B‐cell‐specific molecules, has changed the treatment landscape for high‐risk B‐ALL [2]. In contrast, there are few novel therapeutic options for T‐ALL, as no clinically effective immunotherapies or molecular targeted therapies are currently available.

In addition to these therapies, differentiation therapy is another option for the treatment of leukemia. Differentiation therapy is a less cytotoxic yet effective treatment that induces the terminal differentiation of immature leukemic cells. Notably, the introduction of all‐trans retinoic acid (ATRA) and arsenic trioxide (ATO) has significantly improved treatment outcomes for patients with acute promyelocytic leukemia (APL) [3, 4]. However, no effective differentiation therapy has been established for patients with ALL.

Krüppel‐like factor 4 (KLF4) is a member of the KLF family of transcription factors that regulates various cellular processes, including proliferation and differentiation [5, 6]. *KLF4* expression is silenced by promoter methylation in T‐ALL cells, and the loss of KLF4 promotes the development of NOTCH1‐induced T‐ALL by activating the MAP2K7 pathway [7]. Genetic or pharmacological induction of KLF4 inhibits the proliferation of TAL1‐positive T‐ALL cells by downregulating *TAL1* expression through the suppression of *TAL1* super‐enhancer activity [8]. Although such studies suggest that KLF4 acts as a tumor suppressor in T‐ALL, it remains unclear whether KLF4 regulates the differentiation of T‐ALL cells.

In the present study, we demonstrated that KLF4 promoted T‐cell differentiation in TALL‐1 cells harboring an activating mutation in NOTCH3 by directly downregulating *NOTCH3* expression. Furthermore, the pharmacological induction of KLF4 promoted T‐cell differentiation and exhibited anti‐leukemic activity in TALL‐1 cells. These findings suggest a promising differentiation therapy for T‐ALL with activating mutations in NOTCH3.

## 2 Materials and Methods

### 2.1 Cell Lines

Human T‐ALL cell lines (Jurkat, MOLT‐3, CCRF‐CEM, RPMI‐8402, HPB‐ALL, and TALL‐1) were cultured in Roswell Park Memorial Institute 1640 medium (Wako, Osaka, Japan) containing 10% fetal bovine serum (FBS; Thermo Fisher Scientific, USA) and 1% penicillin–streptomycin (PS; Wako) under 5% CO_2_ and 95% air at 37°C. Human embryonic kidney (HEK) 293 T cells were maintained in Dulbecco's modified Eagle's medium (Wako) supplemented with 10% FBS and 1% PS in a humidified incubator with 5% CO_2_ and 95% air at 37°C.

### 2.2 Reagents

APTO‐253 was purchased from MedChemExpress, USA. DAPT was obtained from AdipoGen Life Sciences, USA. 10058‐F4 was purchased from Abcam, USA.

### 2.3 Expression Plasmids

Human *KLF4* cDNA was amplified by PCR and inserted into the pENTR1A dual selection vector (Thermo Fisher Scientific, USA) and CSIV‐TRE‐Ubc‐KT expression vectors. CSIV‐TRE‐Ubc‐KT was kindly provided by Dr. H. Miyoshi (RIKEN BRC, Japan).

### 2.4 Lentivirus Production and Transduction

HEK293T cells were transiently co‐transfected with lentiviral vectors, psPAX2, and pMD2.G using PEI Max (MW 40000) (Polysciences, USA). Then, 48 h after transfection, viral supernatants were collected and used immediately for infection. Successfully transduced cells were sorted using a FACSAria II Cell Sorter (BD Biosciences, USA).

### 2.5 Immunoblotting

Cells were washed with PBS and lysed in RIPA buffer (Wako). After centrifugation, the protein content of the supernatants was measured using the DC Protein Assay (Bio‐Rad Laboratories, USA). Equal amounts of whole‐cell lysates were separated by SDS/PAGE and electrotransferred onto polyvinylidene difluoride membranes. The membranes were probed with the following primary antibodies at a dilution of 1:1000: anti‐GAPDH (0411; Santa Cruz Biotechnology, USA), anti‐KLF4 (#4038; Cell Signaling Technology, USA), and anti‐Notch3 (#2889; Cell Signaling Technology). Horseradish peroxidase (HRP)‐conjugated anti‐rabbit IgG and anti‐mouse IgG (Cell Signaling Technology) were used as secondary antibodies at a dilution of 1:5000. The blots were visualized using ECL Prime Western Blotting Detection Reagent (Cytiva, Japan) and Light‐Capture II (ATTO, Japan) according to the manufacturer's recommendations.

### 2.6 Reverse Transcription‐Quantitative Polymerase Chain Reaction (RT‐qPCR)

Total RNA was isolated using the ReliaPrep RNA Cell Miniprep System (Promega, USA) and reverse‐transcribed using a reverse‐script kit (TOYOBO, Japan) to generate cDNA. RT‐qPCR was performed on the Thermal Cycler Dice Real‐Time System II (Takara Bio, Japan) according to the manufacturer's recommendations. The results were normalized to the expression of *glyceraldehyde‐3‐phosphate dehydrogenase* (*GAPDH*). The relative expression levels were calculated using the 2^−ΔΔCt^ method [9]. Primers used for RT‐qPCR are listed in Table S1.

### 2.7 Chromatin Immunoprecipitation‐Quantitative Polymerase Chain Reaction (ChIP‐PCR)

The ChIP assay was performed as previously described [8] using an anti‐KLF4 antibody (#12173, 1:100 dilution; Cell Signaling Technology). The primer sequences used for PCR are listed in Table S2.

### 2.8 shRNA Interference

Specific short‐hairpin RNAs (shRNAs) targeting KLF4 and NOTCH3 were initially subcloned into pENTR4‐H1tetOx1 vectors, with the resulting shKLF4 and shNOTCH3 constructs subsequently being introduced into the vectors CS‐RfA‐ETBsd and CS‐RfA‐ETV, respectively, which were generously provided by Dr. H. Miyoshi (RIKEN BRC, Japan). As a non‐targeting control, we also designed an shRNA against luciferase (sh_Luc). The sequences targeted by the shRNAs are listed in Table S3.

### 2.9 Flow Cytometry

T‐cell differentiation was assessed using APC anti‐human CD3 Antibody (344 812; BioLegend, USA), APC anti‐human CD4 Antibody (317 416; BioLegend), and PerCP/Cyanine5.5 anti‐human CD8 Antibody (344 710; BioLegend) at a dilution of 1:20. Flow cytometry was performed using a FACSCalibur and FACSAria II (BD Biosciences), and data analysis was performed using FlowJo software (BD Biosciences).

### 2.10 Apoptosis Assay

The cells were washed in PBS, suspended in 100 μL of annexin V binding buffer, and then mixed with 5 μL of annexin V (BioLegend). The reaction mixture was incubated for 15 min. After incubation, the cells were diluted and processed for flow cytometry.

### 2.11 Statistical Analysis

Differences between the control and experimental groups were assessed using a 2‐tailed unpaired Student's *t*‐test and were considered significant if the *p‐*value was less than 0.05. The equality of variance between the two populations was calculated using an *F*‐test. The results are presented as the mean ± standard error of the mean (SEM) of the values obtained from three independent experiments.

### 2.12 Study Approval

We did not perform any experiments involving humans or animals in this study.

## 3 Results

### 3.1 KLF4 Induced T‐Cell Differentiation in TALL‐1 Cells

**FIGURE 1:**
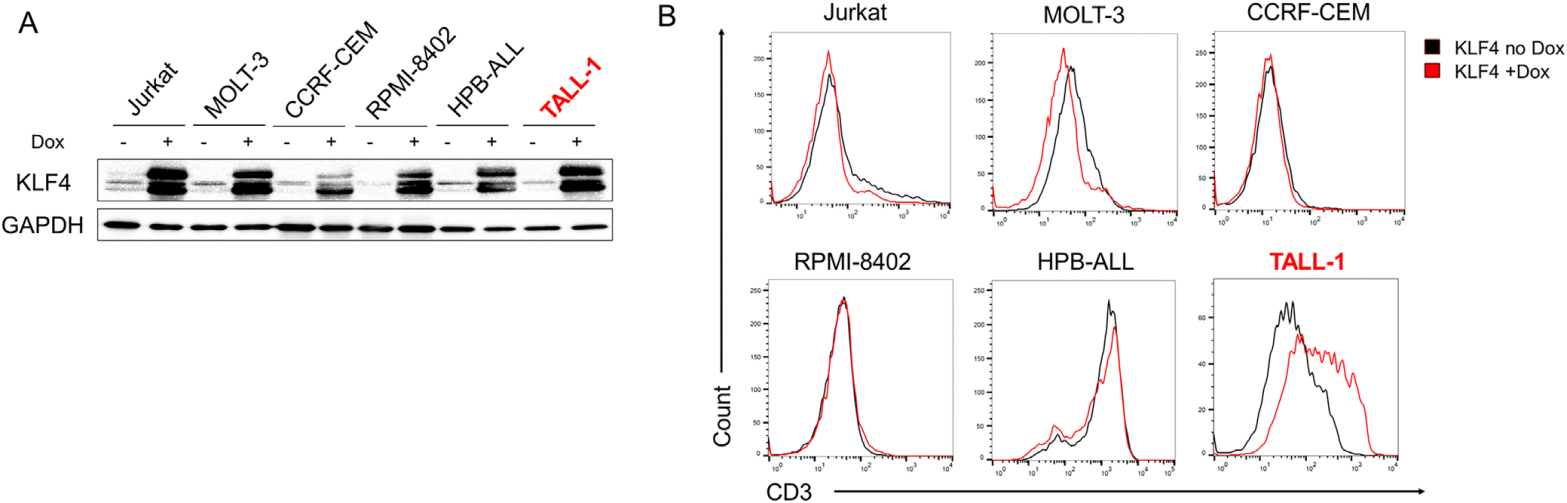
KLF4 increased surface CD3 expression on TALL‐1 cells. (A) Immunoblot analysis of KLF4 in Dox‐treated Di‐KLF4 cell lines. The cells were treated with or without 3 μM doxycycline (Dox) for 48 h and then lysed for protein extraction. (B) Surface CD3 expression on Dox‐treated Di‐KLF4 T‐ALL cell lines. The cells were treated with or without 3 μM Dox for 3–6 days.

**FIGURE 2:**
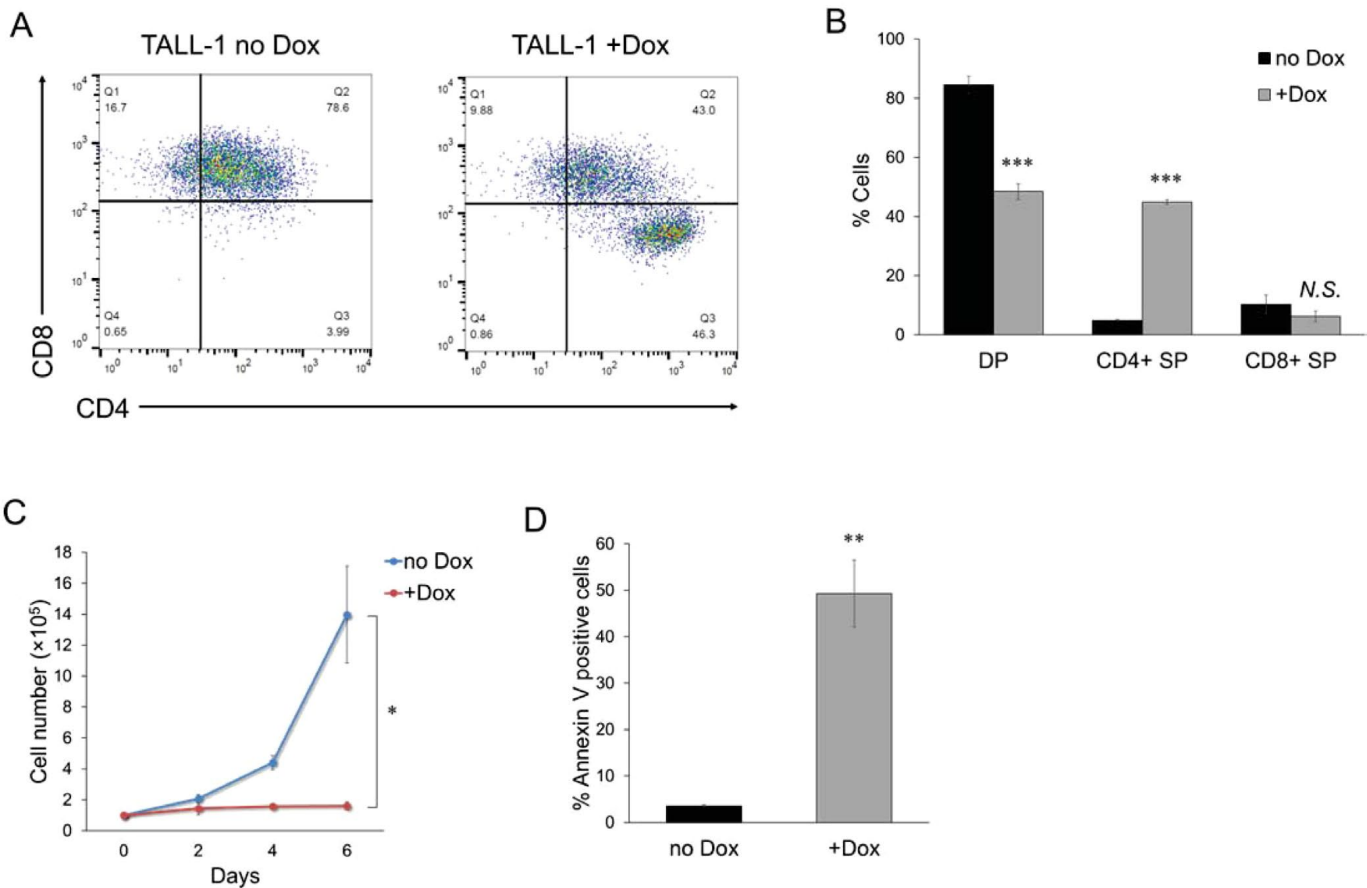
KLF4 induced T‐cell differentiation in TALL‐1 cells. (A and B) KLF4 overexpression induced the differentiation of CD4/CD8 double‐positive (DP) cells into CD4 single‐positive (SP) cells in TALL‐1 cells. The cells were treated with or without 3 μM Dox for 6 days (*n* = 3). (C) Growth inhibition by KLF4 in Di‐KLF4/TALL‐1 cells. To assess cell proliferation, 1 × 10^5^ cells were seeded in a 6‐well plate. The cells were cultured with or without 3 μM Dox (*n* = 3). Trypan blue dye exclusion assay was performed every other day. (D) Apoptosis induced by KLF4 in Di‐KLF4/TALL‐1 cells. The cells were cultured with or without 3 μM Dox for 6 days, and then the annexin V‐positive cells were scored by flow cytometric analysis (*n* = 3). Data are presented as the mean ± SEM. *p < 0.05, **p < 0.01, ***p < 0.001, N.S., not significant, as determined using a two‐tailed Student's *t‐*test (B–D).

To investigate whether KLF4 induces T‐cell differentiation in T‐ALL cell lines, we lentivirally transduced a Doxycycline (Dox)‐inducible KLF4 expression vector into six T‐ALL cell lines (Jurkat, MOLT‐3, CCRF‐CEM, RPMI‐8402, HPB‐ALL, and TALL‐1), establishing Dox‐inducible (Di)‐KLF4 T‐ALL cell lines. KLF4 expression in these cells was induced by treatment with 3 μM Dox (Figure 1A). We first examined the surface expression of CD3, a pan‐T‐cell marker typically found on mature T cells. As shown in Figure 1B, surface CD3 expression increased only in Dox‐treated Di‐KLF4/TALL‐1 cells. Therefore, we focused on TALL‐1 and investigated whether KLF4 promotes the differentiation of TALL‐1, a CD4/CD8 double‐positive (DP) cell line [10]. KLF4 overexpression significantly decreased the DP cell population and increased the CD4 single‐positive (SP) cell population (Figure 2A,B). Although KLF4 overexpression caused a slight increase in the CD4 SP cell population in HPB‐ALL, another CD4/CD8 DP cell line [10], this effect was not as significant as that observed in TALL‐1 (Figure S1). KLF4 overexpression inhibited proliferation and induced apoptosis of TALL‐1 cells (Figure 2C,D). These results suggest that KLF4 exerts an anti‐leukemic effect on TALL‐1 cells by promoting T‐cell differentiation and subsequent apoptosis.

### 3.2 KLF4 Directly Suppressed *NOTCH3* Transcription in TALL‐1 Cells

**FIGURE 3:**
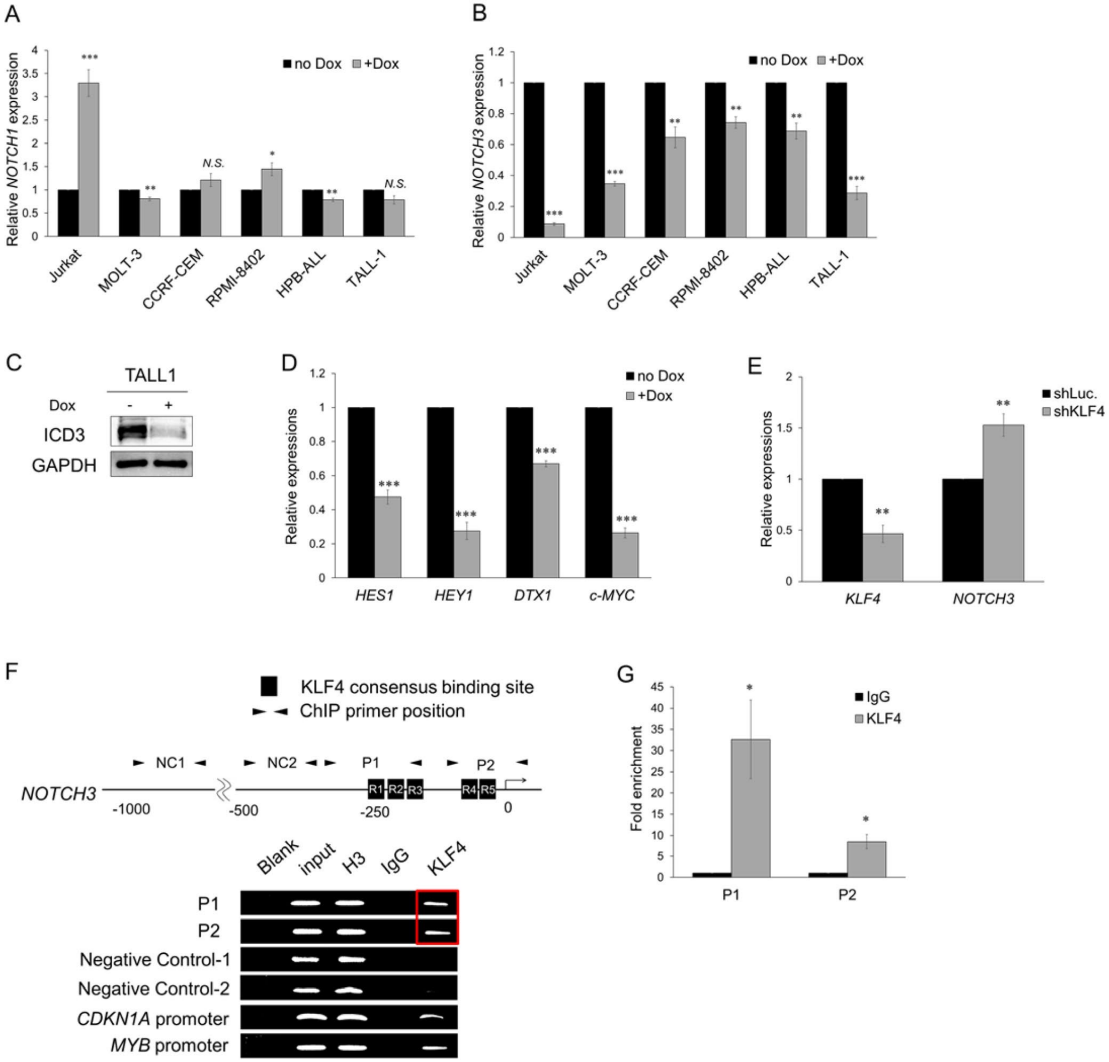
KLF4 directly downregulated *NOTCH3* expression in TALL‐1 cells. (A) Relative mRNA expression levels of *NOTCH1* in Di‐KLF4 cell lines. The cells were treated with or without 3 μM Dox for 48 h, and then total RNA was prepared and analyzed by RT‐qPCR. Values were normalized to the expression levels of *GAPDH* (*n* = 3). (B) Relative mRNA expression levels of *NOTCH3* in Di‐KLF4 cell lines. The cells were treated as described in (A) (*n* = 3). (C) Immunoblot analysis of the intracellular domain of NOTCH3 (ICD3) in Di‐KLF4/TALL‐1 cells. The cells were treated with or without 3 μM Dox for 48 h and then lysed for protein extraction. (D) Relative mRNA expression levels of NOTCH target genes (*HES1, HEY1, DTX1, c‐MYC*) in Di‐KLF4/TALL‐1 cells. The cells were treated as described in (A) (*n* = 3). (E) Relative mRNA expression levels of *KLF4* and *NOTCH3* following the knockdown of KLF4 or control luciferase (Luc.) in Jurkat cells. The cells were treated with or without 3 μM Dox for 48 h, and then total RNA was prepared and analyzed by RT‐qPCR. Values were normalized to the expression levels of *GAPDH* (*n* = 3). (F) KLF4 bound to the *NOTCH3* promoter. The upper image shows the proximal regulatory region of *NOTCH3*. The lower image shows the results of the ChIP analysis of Di‐KLF4/TALL‐1 cells. The cells were treated with or without Dox for 48 h to induce KLF4 expression before the ChIP assay. Blank (distilled water only); input DNA; H3 (positive control), IgG (negative control), and KLF4 precipitated reactions were amplified with the indicated primer sets. As negative controls, we used Negative Control‐1 (NC1) and Negative Control‐2 (NC2), which are regions within −1000 to 0 bp relative to the transcription start site that do not include a KLF4 consensus binding site. As positive controls, we used the promoters of *CDKN1A* and *MYB*, both of which have been reported to be direct transcriptional targets of KLF4 [7, 8]. (G) ChIP fold enrichment of DNA fragments precipitated with the anti‐KLF4 antibody relative to those precipitated with normal IgG, as determined by qPCR using the primer sets indicated in (F) (*n* = 3). Data are presented as the mean ± SEM. *p < 0.05, **p < 0.01, ***p < 0.001, N.S., not significant, as determined using a two‐tailed Student's *t‐*test (A, B, D, E, G).

Given that KLF4 is a transcription factor, we speculated that KLF4 promotes T‐cell differentiation in TALL‐1 cells by transcriptionally regulating the genes essential for this process. While all five T‐ALL cell lines, which did not undergo T‐cell differentiation upon KLF4 overexpression, have activating mutations in NOTCH1, the *NOTCH1* gene in TALL‐1 cells is wild‐type [10, 11]. Instead, TALL‐1 harbors an activating NOTCH3 mutation in the negative regulatory region domain, leading to ligand‐independent NOTCH3 activation [11]. In normal thymocytes, NOTCH3 is expressed at the double‐negative (DN) and DP stages and decreases after the DN to DP transition [12]. Using the BloodSpot database [13], we found that *NOTCH3* expression is significantly higher in patients with T‐ALL than in healthy individuals (Figure S2A). In addition, at both the mRNA and protein levels, the expression of NOTCH3 was found to be significantly higher in TALL‐1 cells than in other cell lines (Figure S2B,C). Based on these findings, we hypothesized that downregulation of NOTCH3 during the DP stage is essential for differentiation into the SP stage, and that KLF4 induces T‐cell differentiation in TALL‐1 cells by transcriptionally repressing *NOTCH3* expression. KLF4 overexpression had variable effects on *NOTCH1* mRNA levels in Di‐KLF4 cell lines, increasing them in Jurkat and RPMI‐8402 cells, and causing a slight decrease in MOLT‐3 and HPB‐ALL cells (Figure 3A). On the other hand, KLF4 overexpression significantly decreased *NOTCH3* expression in all Di‐KLF4 cell lines (Figure 3B). The protein levels of the NOTCH3 intracellular domain (ICD3) were reduced in TALL‐1 cells upon KLF4 overexpression (Figure 3C). Moreover, KLF4 overexpression significantly suppressed the expression of NOTCH target genes, including *HES1*, *HEY1*, *DTX1*, and *c‐MYC* (Figure 3D). To further confirm the specificity of KLF4 for NOTCH3, we generated a Dox‐inducible shRNA targeting KLF4 and lentivirally transduced it into Jurkat cells. In line with expectations, we found that the knockdown of KLF4 promoted a significant upregulation of *NOTCH3* expression (Figure 3E). Analysis of the proximal *NOTCH3* promoter region revealed five KLF4 consensus binding sites (R1‐R5) within −250 to 0 bp relative to the transcription start site. ChIP assay using an anti‐KLF4 antibody confirmed the direct binding of KLF4 to these sites (Figure 3F,G), indicating that KLF4 directly regulates *NOTCH3* expression.

### 3.3 Inhibition of the NOTCH Signaling Pathway Promoted T‐Cell Differentiation in TALL‐1 Cells

**FIGURE 4:**
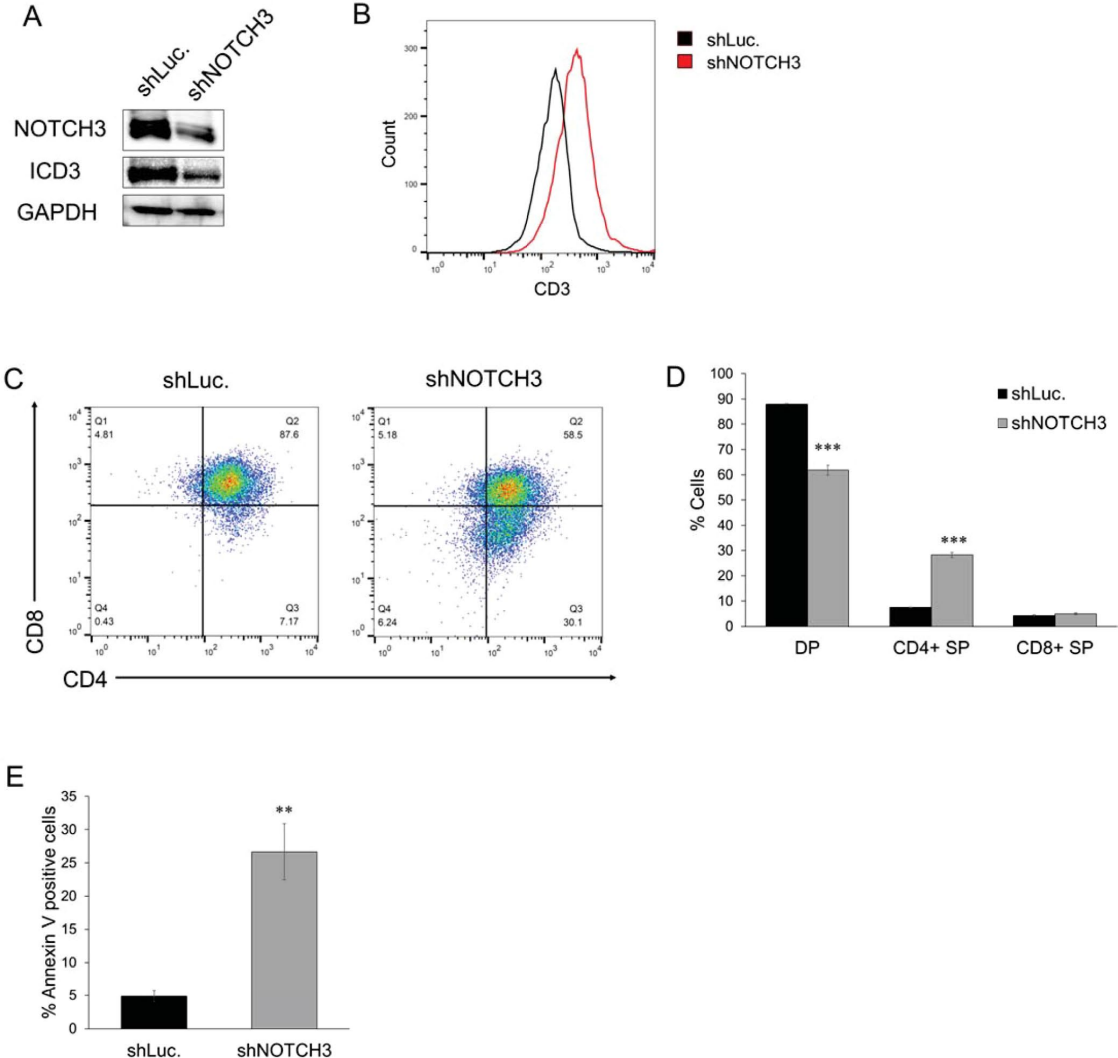
NOTCH3 inhibition induced T‐cell differentiation in TALL‐1 cells. (A) Immunoblot analysis of NOTCH3 and ICD3 following NOTCH3 knockdown in TALL‐1 cells. The cells were treated with or without 3 μM Dox for 48 h and then lysed for protein extraction. (B) Surface CD3 expression on TALL‐1 cells following NOTCH3 knockdown. The cells were treated with or without 3 μM Dox for 8 days. (C, D) NOTCH3 knockdown induced the differentiation of CD4/CD8 DP cells into CD4 SP cells in TALL‐1 cells. The cells were treated as described in (B) (*n* = 3). (E) Apoptosis induced by NOTCH3 knockdown in TALL‐1 cells. The cells were treated as described in (B), and then the annexin V‐positive cells were scored by flow cytometric analysis (*n* = 4). Data are presented as the mean ± SEM. **p < 0.01, ***p < 0.001, as determined using a two‐tailed Student's *t‐*test (D, E).

**FIGURE 5:**
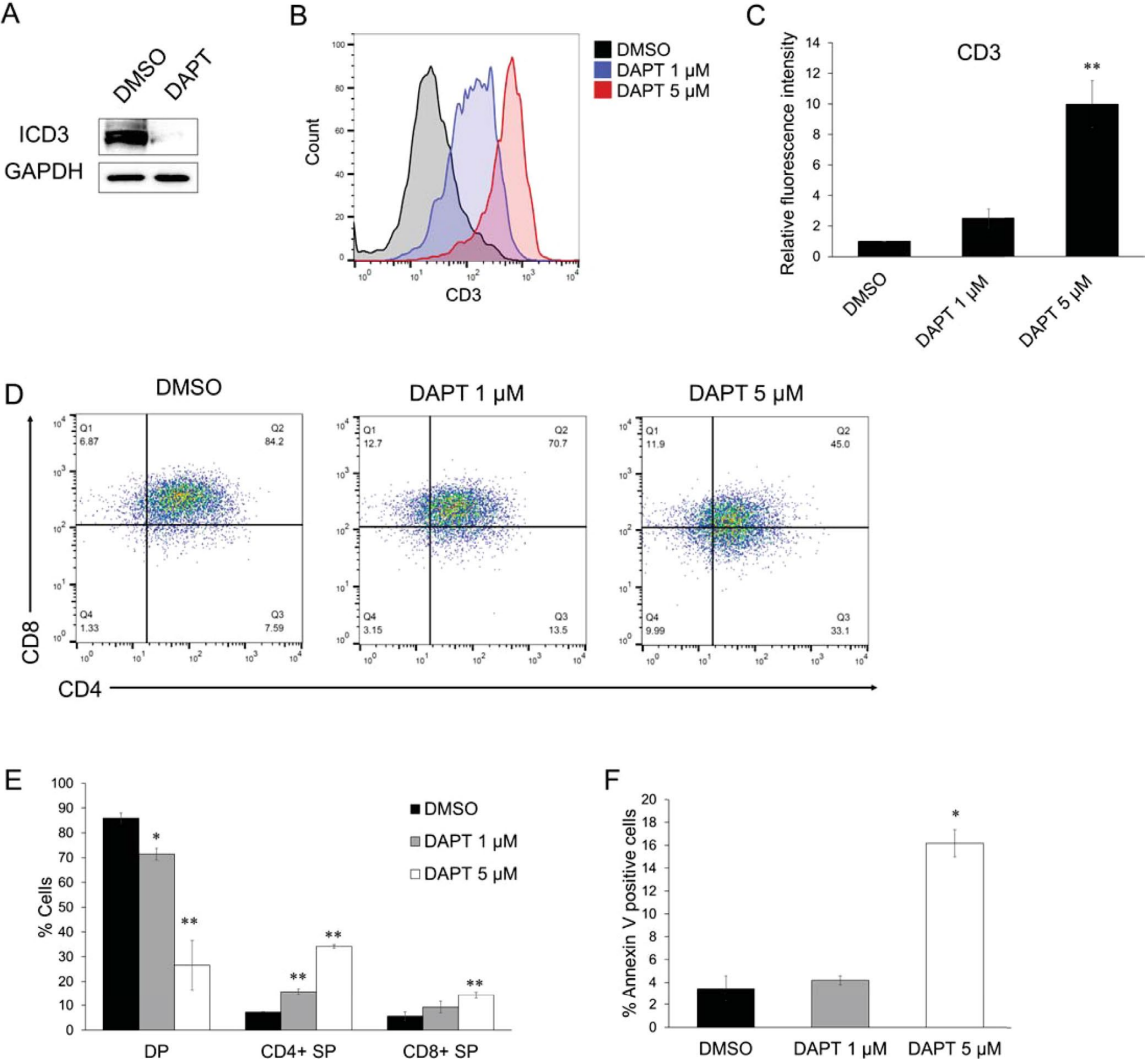
DAPT promoted T‐cell differentiation in TALL‐1 cells. (A) Downregulation of ICD3 expression by DAPT treatment in TALL‐1 cells. The cells were cultured with DMSO or 5 μM DAPT for 72 h and then lysed for protein extraction. (B, C) DAPT increased surface CD3 expression on TALL‐1 cells. The cells were treated with DMSO or the indicated concentration of DAPT for 6 days (*n* = 3). (D, E) DAPT induced the differentiation of CD4/CD8 DP cells into CD4 SP cells. The cells were treated as described in (B) (*n* = 3). (F) Apoptosis induced by treatment with DAPT in TALL‐1 cells. The cells were treated as described in (B), and then the annexin V‐positive cells were scored by flow cytometric analysis (*n* = 3). Data are presented as the mean ± SEM. *p < 0.05, **p < 0.01, as determined using a two‐tailed Student's *t‐*test (C, E, F).

To confirm that KLF4‐induced T‐cell differentiation is due to the downregulation of *NOTCH*3 and the subsequent reduction in ICD3 expression, we investigated whether inhibition of the NOTCH signaling pathway induces T‐cell differentiation in TALL‐1 cells. We generated a Dox‐inducible shRNA targeting NOTCH3 and lentivirally transduced it into TALL‐1 cells. NOTCH3 knockdown effectively reduced the levels of both NOTCH3 and ICD3 proteins in TALL‐1 cells (Figure 4A). NOTCH3 knockdown increased the surface expression of CD3 (Figure 4B) and promoted the differentiation of CD4/CD8 DP cells into CD4 SP cells (Figure 4C,D), followed by apoptosis of the differentiated TALL‐1 cells (Figure 4E). To pharmacologically inhibit NOTCH signaling, we treated TALL‐1 cells with DAPT, a γ‐secretase inhibitor (GSI) known to suppress NOTCH3 and ICD3 expression in TALL‐1 cells [11]. DAPT treatment efficiently reduced ICD3 protein levels in TALL‐1 cells (Figure 5A). Treatment with 5 μM DAPT for 6 days significantly increased the surface expression of CD3 (Figure 5B,C) and promoted the differentiation of DP cells primarily into CD4 SP cells (Figure 5D,E). These differentiated cells subsequently underwent apoptosis (Figure 5F). We further examined whether DAPT induces T‐cell differentiation in HPB‐ALL, a CD4/CD8 DP cell line with an activating NOTCH1 mutation. Although treatment with DAPT for 6 days moderately increased the surface expression of CD3, it did not promote the differentiation of DP cells into SP cells (Figure S3). These results suggest that inhibition of NOTCH3 signaling, rather than NOTCH1 signaling, is important for T‐ALL cell differentiation.

### 3.4 Pharmacological Induction of KLF4 Effectively Promoted T‐Cell Differentiation in TALL‐1 Cells

**FIGURE 6:**
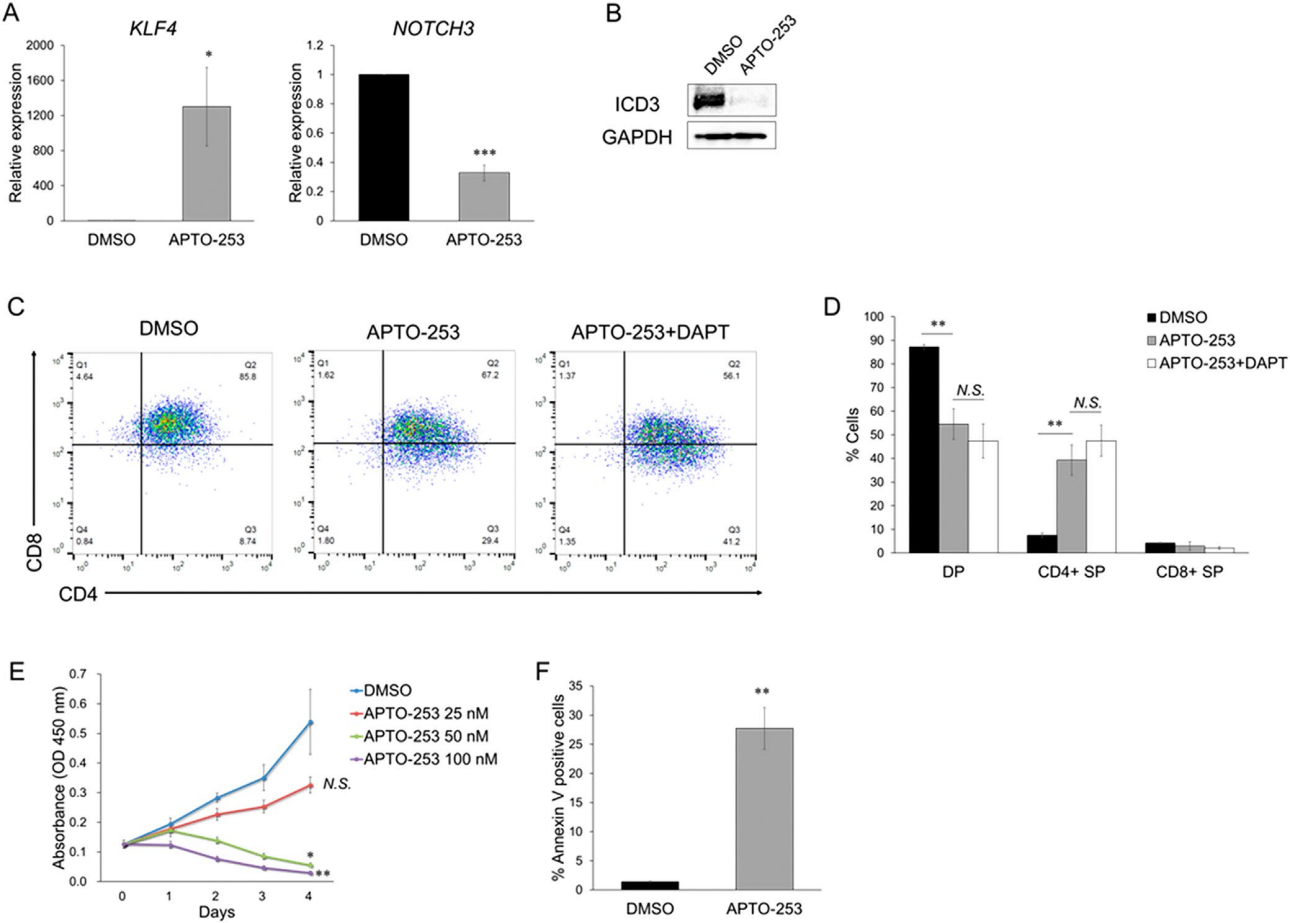
APTO‐253 efficiently promoted T‐cell differentiation in TALL‐1 cells. (A) Upregulation of *KLF4* and downregulation of *NOTCH3* in TALL‐1 cells treated with APTO‐253. The cells were cultured with DMSO or 50 nM of APTO‐253 for 72 h, and then total RNA was prepared and analyzed by RT‐qPCR. Values were normalized to the expression levels of *GAPDH* (*n* = 3). (B) Downregulation of ICD3 expression by APTO‐253 treatment in TALL‐1 cells. The cells were treated as described in (A) and then lysed for protein extraction. (C, D) APTO‐253, both alone and in combination with DAPT, induced the differentiation of CD4/CD8 DP cells into CD4 SP cells. The cells were treated with DMSO, 50 nM APTO‐253 alone, or 50 nM APTO‐253 with 5 μM DAPT for 72 h (*n* = 3). (E) Growth inhibition by APTO‐253 in TALL‐1 cells. To assess cell proliferation, 1 × 10^4^ cells were seeded in a 96‐well plate. The cells were cultured with DMSO or the indicated concentration of APTO‐253 (*n* = 3). Cell proliferation was assessed every 24 h by the WST assay. (F) Apoptosis induced by treatment with APTO‐253 in TALL‐1 cells. The cells were treated as described in (A), and then the annexin V‐positive cells were scored by flow cytometric analysis (*n* = 3). Data are presented as the mean ± SEM. *p < 0.05, **p < 0.01, ***p < 0.001, N.S., not significant, as determined using a two‐tailed Student's *t‐*test (A, D, E, F).

Finally, we investigated whether the induction of KLF4 using APTO‐253 [14, 15], a small‐molecule inducer of KLF4, induces T‐cell differentiation and subsequent apoptosis in TALL‐1 cells. APTO‐253 treatment induced *KLF4* expression and reduced *NOTCH3* expression (Figure 6A), leading to decreased ICD3 protein levels (Figure 6B). APTO‐253 promoted the differentiation of CD4/CD8 DP cells into CD4 SP cells in TALL‐1 cells (Figure 6C,D). When combined with DAPT, APTO‐253 increased the ratio of CD4 SP cells more than APTO‐253 alone did; however, the effect was not synergistic (Figure 6C,D). TALL‐1 cells treated with APTO‐253 showed a dose‐dependent decrease in proliferation (Figure 6E). Furthermore, the differentiated cells underwent rapid apoptosis (Figure 6F). Since APTO‐253 is also a c‐MYC inhibitor [15], we investigated whether c‐MYC inhibition affects the differentiation of TALL‐1 cells. Although treatment with 10 058‐F4, a representative c‐MYC inhibitor, suppressed c‐MYC expression in TALL‐1 cells, it did not promote differentiation of DP cells into SP cells (Figure S4). These results indicated that APTO‐253 effectively promoted T‐cell differentiation and suppressed the growth of TALL‐1 cells through the induction of KLF4.

## 4 Discussion

In the present study, we investigated the role of KLF4 in the differentiation of T‐ALL cells. KLF4 promoted T‐cell differentiation, particularly in TALL‐1 cells harboring an activating NOTCH3 mutation. KLF4 directly downregulated *NOTCH3* expression by binding to its promoter, thereby promoting the differentiation of immature DP T cells into SP cells. In addition, the pharmacological induction of KLF4 using APTO‐253 showed anti‐leukemic activity. APTO‐253 effectively downregulated *NOTCH3* expression and induced the differentiation of T‐ALL cells. Critically, the differentiated cells underwent rapid apoptosis.

Mutations in NOTCH3 are relatively rare in T‐ALL compared to those in NOTCH1. Previous research found NOTCH3 mutations in approximately 1% of pediatric and young adult T‐ALL cases [16]. However, the precise frequency of these mutations remains uncertain because the high G‐C content of NOTCH3, reaching up to 88%, has resulted in limited sequencing depth [11, 17]. Therefore, NOTCH3 mutations in T‐ALL may be underestimated. *NOTCH3* gene expression has been reported in most patients with T‐ALL, whereas it is absent in normal peripheral T cells or non‐T‐cell leukemias [18]. Indeed, our analysis of a public dataset from the Gene Expression Omnibus revealed that *NOTCH3* is overexpressed in most patients with T‐ALL compared to healthy individuals (Figure S2A). Owing to its structure, NOTCH3 exhibits higher basal signaling activity than NOTCH1 and NOTCH2 and is more susceptible to spontaneous activation [19]. Additionally, a previous study showed that some T‐ALL PDX samples without NOTCH3 mutations exhibited NOTCH3 activation [11]. These findings suggest that NOTCH3 overexpression may activate NOTCH3 signaling independently of ligands or mutations. Therefore, direct inhibition of *NOTCH3* expression by KLF4 induction may be an effective therapeutic strategy for T‐ALL with either activating NOTCH3 mutations or NOTCH3 overexpression. Although KLF4 suppressed *NOTCH3* expression in all T‐ALL cell lines we tested (Figure 3B), it induced differentiation only in TALL‐1 cells. We speculate that this is because endogenous *NOTCH3* expression in other cell lines is much lower than that in TALL‐1 cells (Figure S2B,C), and NOTCH1 mutations in these cell lines compensate for impaired NOTCH3 function. Therefore, we propose that KLF4 induction could be effective in T‐ALL cases with active NOTCH3 signaling and without NOTCH1 mutations.

NOTCH3 is an attractive therapeutic target because its selective inhibition is expected to have lower toxicity owing to its more tissue‐specific expression compared to other NOTCH receptors [11]. Indeed, in mouse and rat models, it has been demonstrated that treatment of pulmonary hypertension with an anti‐NOTCH3 antibody (Ab 28 042), which specifically binds to NOTCH3 and inhibits JAGGED1‐induced NOTCH3 cleavage, has no evident toxic side effects [20]. Although to date there have been no studies that have specifically examined the efficacy of Ab 28 042 in the treatment of T‐ALL, we believe this could represent a promising therapeutic strategy, given that the NOTCH3/JAGGED1 auto‐sustaining loop has been reported to play a role in the development and progression of NOTCH‐dependent T‐ALL [21].

In this study, we demonstrated that APTO‐253, a small‐molecule inducer of KLF4, acted as a NOTCH3 inhibitor and promoted the differentiation of T‐ALL cells. Although GSIs, which are pan‐NOTCH inhibitors, effectively inhibit NOTCH3 signaling by blocking the proteolytic activation of NOTCH receptors [22], their anti‐leukemic effects in clinical trials were poor and transient [23]. In addition, GSI treatment is often associated with severe intestinal toxicity, causing diarrhea, vomiting, and nausea due to the simultaneous inhibition of NOTCH1 and NOTCH2 in gut epithelial stem cells [24, 25]. On the other hand, APTO‐253 was well‐tolerated and showed favorable pharmacokinetics in phase I clinical studies on solid tumors (NCT123226) [26] and relapsed or refractory acute myeloid leukemia or high‐risk myelodysplastic syndromes (NCT02267863) [27]. Previously, we demonstrated that APTO‐253 effectively exerted anti‐leukemic effects on TAL1‐positive T‐ALL cell lines in vitro and in vivo [8]. However, its ability to promote the differentiation of T‐ALL cells remained unclear. While differentiation therapy using ATRA and ATO has been highly successful in treating APL, no such approach has yet been established for patients with ALL. In this context, we demonstrated that targeting NOTCH3 with APTO‐253 may represent a promising differentiation therapy for certain subsets of patients with T‐ALL. Furthermore, we speculate that APTO‐253 may also target other diseases associated with NOTCH3 mutations or overexpression, including solid tumors, such as head and neck squamous cell carcinoma, ovarian cancer, and lung adenocarcinoma [11], as well as cerebral autosomal dominant arteriopathy with subcortical infarcts and leukoencephalopathy (CADASIL) [28] and pulmonary arterial hypertension [20].

Although this study successfully demonstrated that KLF4 promoted T‐cell differentiation in TALL‐1 cells by downregulating NOTCH3 expression, we failed to confirm this in other cell lines because none of them harbored activating NOTCH3 mutations. Further experiments using clinical samples with activating NOTCH3 mutations or NOTCH3 overexpression are required to confirm our findings.

## Author Contributions

M.N. initiated the study, designed and performed the experiments, analyzed the data, and wrote the manuscript. All the other researchers supervised the study and approved the final manuscript for submission.

## Conflicts of Interest

Hitoshi Kiyoi received research funding from FUJIFILM, Kyowa‐Kirin, Bristol‐Myers Squibb, Otsuka, Perseus Proteomics, Daiichi Sankyo, Abbvie, CURED, Astellas Pharma, Chugai, Zenyaku Kogyo, Nippon Shinyaku, Eisai, Takeda, Sumitomo Pharma, and Sanofi, and honoraria from Abbvie, Chugai, Astellas Pharma, and Novartis.

## Supporting information


Data S1:

## Data Availability

The data that support the findings of this study are available from the corresponding author upon reasonable request.
