# Supplementary material for: Induction of T‐Cell Differentiation by KLF4 in T‐Cell Acute Lymphoblastic Leukemia Cells Harboring Activating Mutation in NOTCH3
Source: FASEB J. 2025 May 12;39(10):e70613. doi: 10.1096/fj.202402997R (PMC12068419; doi:10.1096/fj.202402997R)
Supplement: Supplementary file 1 — Data S1: [file FSB2-39-e70613-s001.docx]

**Supplemental Data**

**Induction of T cell differentiation by KLF4 in T-cell acute lymphoblastic leukemia cells harboring activating mutation in NOTCH3**

Mina Noura, Takahiko Yasuda, Hitoshi Kiyoi, Fumihiko Hayakawa.

Supplementary Tables p.2

Supplementary Figures p.5

**Supplemental Table 1**

PCR primers used for RT-qPCR.

**Supplemental Table 2**

PCR primers used for ChIP.

**Supplemental Table 3**

Target sequences for shRNA knockdown experiments.

**
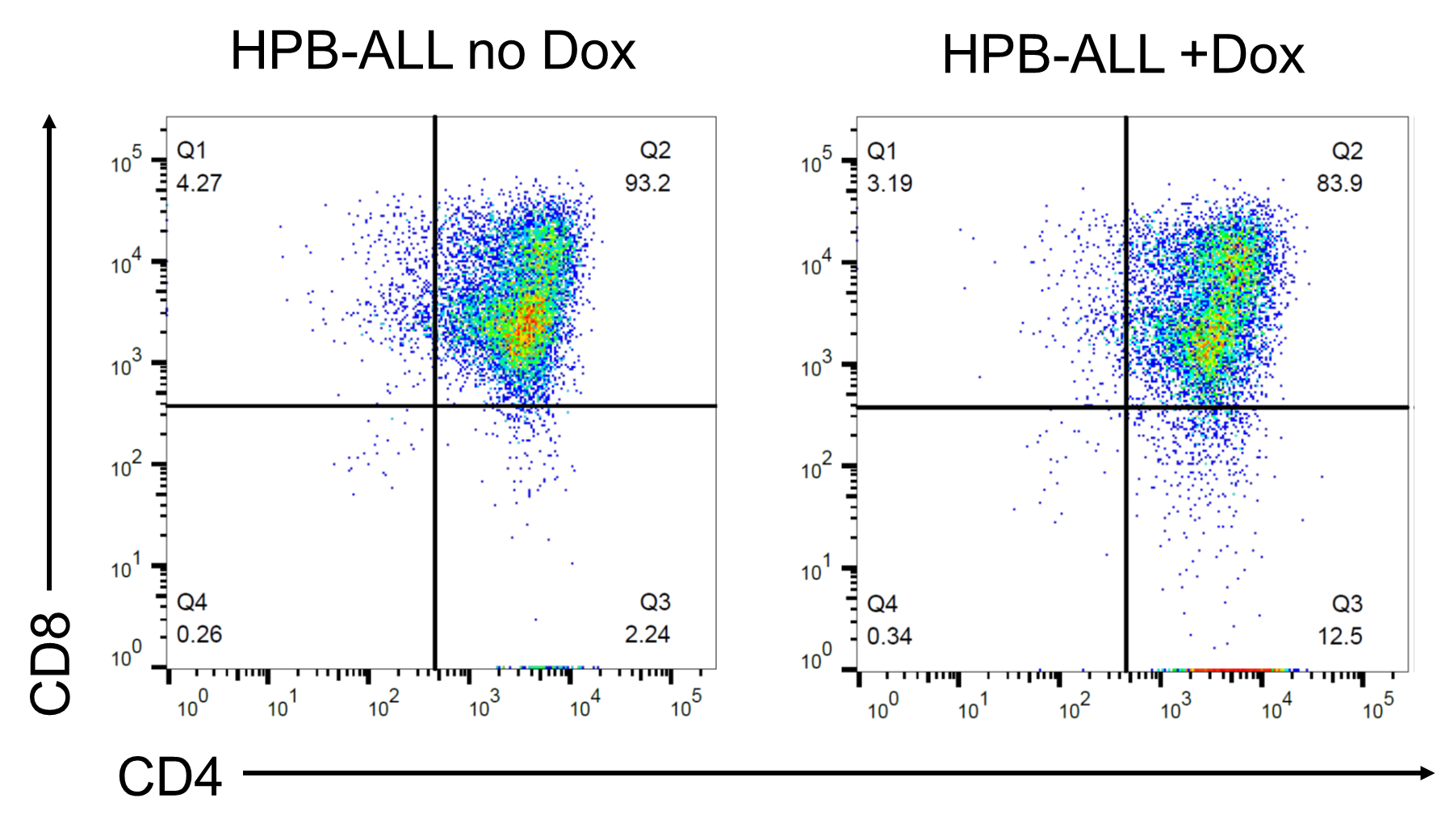
**

**Supplemental Figure 1**

Surface CD4 and CD8 expressions on Di-KLF4/HPB-ALL cells. The cells were treated with or without 3 μM Dox for 6 days.

**
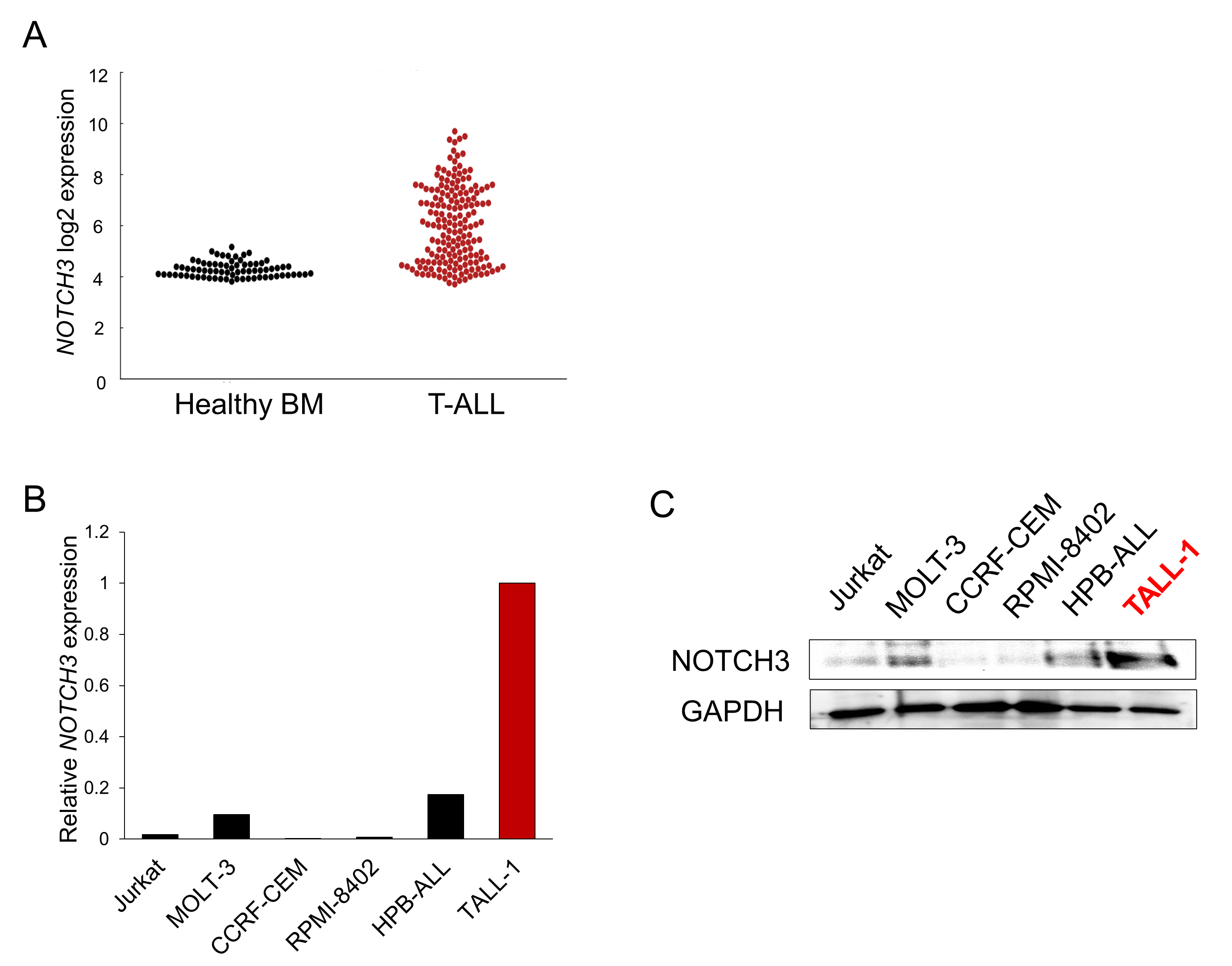
**

**Supplemental Figure 2**

(A) Swarm plot showing the expression levels of the *KLF4* gene in bone marrow samples

from healthy individuals and patients with T-ALL. Data were obtained from the GSE13159 dataset, which includes healthy bone marrow (n = 73) and T-ALL samples (n = 174).

(B) Relative mRNA expression levels of *NOTCH3* in T-ALL cell lines. Total RNA was

prepared and analyzed by RT-qPCR. Values were normalized to the expression levels of *GAPDH*.

(C) Immunoblot analysis of NOTCH3 in T-ALL cell lines.

**
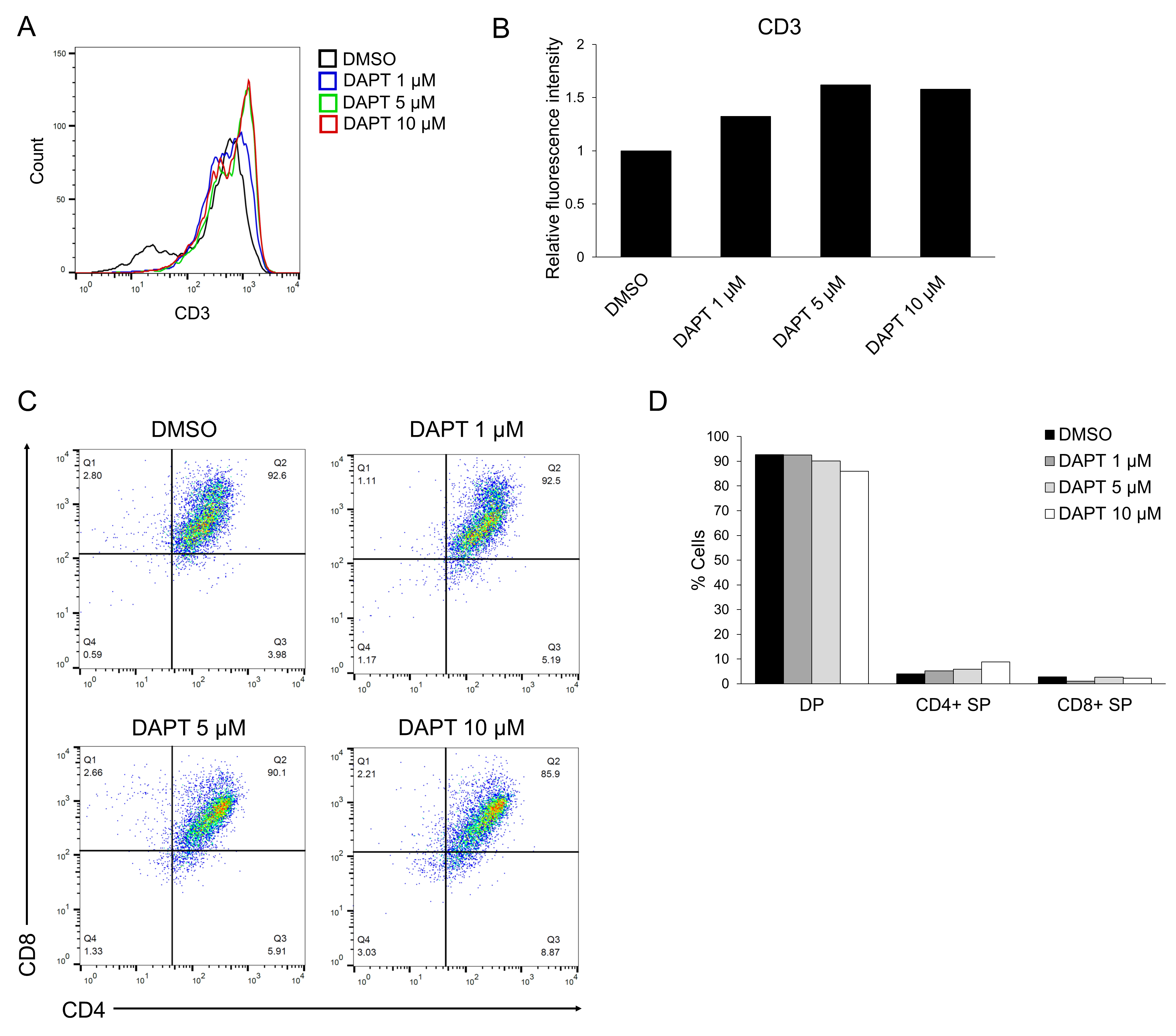
**

**Supplemental Figure 3**

(A and B) Surface CD3 expression on HPB-ALL cells treated with the indicated concentration of DAPT for 6 days.

(C and D) DAPT did not promote the differentiation of HPB-ALL cells. The cells were treated as described in (A).

**
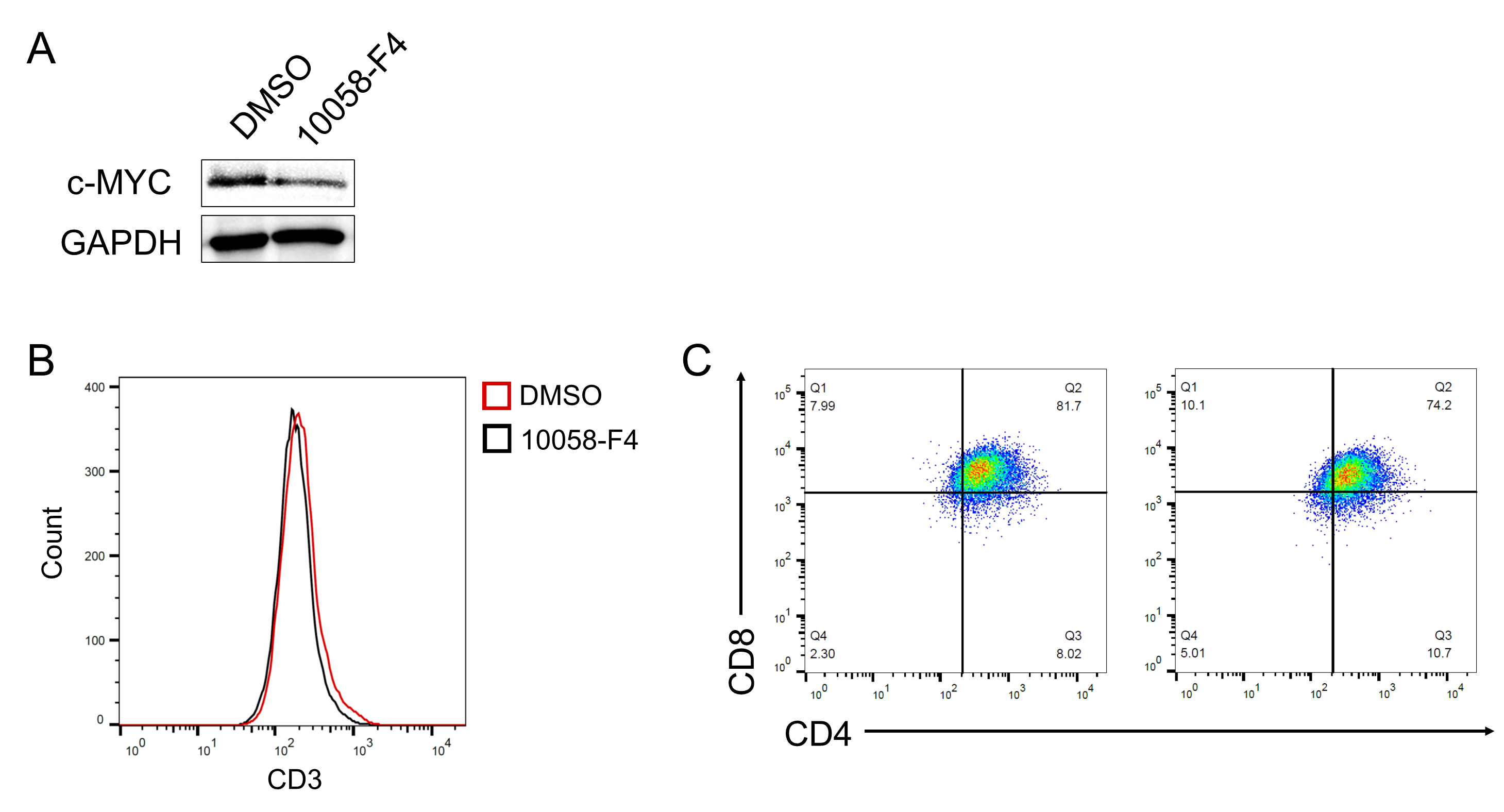
**

**Supplemental Figure 4**

(A) Downregulation of c-MYC expression by 10058-F4 treatment in TALL-1 cells. The cells were treated with DMSO or 50 µM 10058-F4 for 72 h and then lysed for protein extraction.

(B) Surface CD3 expression on TALL-1 cells treated as described in (A).

(C) 10058-F4 did not promote the differentiation of TALL-1 cells. The cells were treated as described in (A).
